# 血友病替代治疗研究新进展

**DOI:** 10.3760/cma.j.issn.0253-2727.2023.12.018

**Published:** 2023-12

**Authors:** 鹏霄 侯, 仁池 杨

**Affiliations:** 1 中国医学科学院血液病医院（中国医学科学院血液学研究所），实验血液学国家重点实验室，国家血液系统疾病临床医学研究中心，细胞生态海河实验室，天津市血液病基因治疗研究重点实验室，中国医学科学院血液病基因治疗重点实验室，天津 300020 State Key Laboratory of Experimental Hematology, National Clinical Research Center for Blood Diseases, Haihe Laboratory of Cell Ecosystem, Institute of Hematology & Blood Diseases Hospital, Chinese Academy of Medical Sciences & Peking Union Medical College, Tianjin Key Laboratory of Gene Therapy for Blood Diseases, CAMS Key Laboratory of Gene Therapy for Blood Diseases, Tianjin 300020, China; 2 天津医学健康研究院，天津 301600 Tianjin Institutes of Health Science, Tianjin 301600, China

血友病是一种罕见的X染色体连锁隐性遗传性出血性疾病，可分为血友病A（HA）和血友病B（HB），两者分别表现为凝血因子Ⅷ（FⅧ）和凝血因子Ⅸ（FⅨ）缺乏，若无及时有效的治疗或干预不但会导致关节畸形，还可能由于重要器官出血而危及生命。血友病基因治疗现已进入后期临床试验阶段并取得突破性成果，但仍面临着诸多问题及挑战。近年来半衰期延长的凝血因子制品、新型旁路制剂及非因子药物层出不穷，其研发及应用极大推进了血友病治疗模式转变。笔者拟从新型凝血因子制品和非凝血因子药物两个方面进行综述，以总结该病国内外最新替代治疗研究进展。

一、凝血因子类药物

长半衰期（EHL）凝血因子通过蛋白（Fc或白蛋白）融合及聚乙二醇化等技术延缓FⅨ或FⅧ在体内的代谢过程，使二者半衰期分别延长3～5倍及1.4～1.8倍，后者倍数的限制与血管性血友病因子（vWF）有关[1]。BIVV001（Efanesoctocog alfa）由单链FⅧ、人类免疫球蛋白IgG1的Fc结构域、2个XTEN多肽及vWF之D′D3结构域组成，其最突出的特征为分子内部FⅧ不受机体内源性vWF影响，加之Fc融合及XTEN多肽的引入使FⅧ半衰期进一步延长[2]–[3]。在Efanesoctocog alfa的早期试验中，测得药物平均半衰期约为40 h，是重组FⅧ（rFⅧ）的3～4倍[4]。另一项重复剂量的Ⅰ期研究以每周1次的频率予Efanesoctocog alfa并连用4周，在用药后3 d观察到受试者平均FⅧ活性高于40％，7 d后仍高于10％[5]。上述研究表明，Efanesoctocog alfa可将患者FⅧ活性维持在一个较高水平且持续较长时间。该药的最新Ⅲ期研究结果已公布，以每周1次的间隔予Efanesoctocog alfa 50 IU/kg预防治疗的受试者（133例）中位年化出血次数（ABR）为0，预计平均ABR不足1[6]。作为目前半衰期最长的EHL FⅧ制品，Efanesoctocog alfa将重型HA患者预防性给药频率延长至每周1次，具有更强的出血保护作用，该药已于今年被美国食品药品监督管理局（FDA）批准用于HA的临床治疗[7]。Efanesoctocog alfa的长期疗效及引发抑制物的风险仍需进一步加以研究。

在我国，rFⅧ产品数量较少且成本较高，作为我国自主研发的首个B结构域缺失（BDD）的第三代rFⅧ产品，Omfiloctocog alfa（SCT800）的出现有助于改善这一现状。一项以HA小鼠为模型的前临床研究显示同已广泛市售的BDD- rFⅧ产品Xyntha®相比，Omfiloctocog alfa具备与之相似的药物代谢动力学（PK）和药效学特征[8]。在一项前瞻性、多中心、开放性、单臂Ⅲ期临床研究中，纳入的12～65岁的重型HA患者（73例）在接受Omfiloctocog alfa预防治疗24周后，平均ABR为2.82，期间89.4％的出血事件在注射1～2次Omfiloctocog alfa后得以控制，抑制物发生率为0，未观察到严重不良反应及过敏反应[9]。该研究表明Omfiloctocog alfa在成人及青少年HA患者出血事件的预防及治疗中是安全且有效的。在一项纳入69例我国重型HA患儿的Ⅲ期临床试验中，接受Omfiloctocog alfa预防性输注的受试者总体ABR为4.05，止血治疗成功率达到92.1％，整体耐受性良好，提示Omfiloctocog alfa同样可用于12岁以下重型HA儿童出血事件的预防及控制[10]。目前一项针对其长期疗效及安全性的试验（NCT03947567）正在进行。

在使用外源性FⅨ及FⅧ的过程中，抑制物的产生给血友病的治疗带来了挑战。作为旁路制剂（BPA）之一，重组活化凝血因子FⅦ（rFⅦa）已被证实在大剂量条件下与活化血小板结合，在其表面直接激活凝血因子Ⅹ（FⅩ）生成足量凝血酶起到止血作用，此途径无需组织因子（TF）、FⅧ、FⅨ[11]。相较于前代产品，第二代rFⅦa药物Eptacog beta结合内皮细胞蛋白C受体（EPCR）能力更强，达到同样止血效能用量更小[11]。Wang等[12]研究了Eptacog beta在伴有抑制物的HA或HB患者中按需治疗的情况，止血方案为应用初始剂量75 µg/kg后每3 h续用75 µg/kg或以225 µg/kg为初始剂量9 h后再续用75 µg/kg，观测到前者和后者在12 h内控制轻中度出血事件的成功率分别为84.9％和93.2％。基于此试验结果，Eptacog beta被FDA批准用于12岁及以上血友病伴抑制物患者出血事件的治疗和控制。此外，一项多中心、随机化的Ⅲ期试验显示Eptacog beta在伴有抑制物的血友病儿童中24 h内止血成功率达90％以上，另一项试验显示Eptacog beta在各年龄段有抑制物的血友病患者手术应用中成功率为81.8％；在多项试验中其耐受性良好，无过敏事件及血栓事件的报道，这些结果为Eptacog beta扩展到儿童人群及围术期管理提供了证据[13]–[15]。另一种新型药物Marzeptacog alfa基于原rFⅦa结构在重链和轻链分别替换两个氨基酸（Q286R和M298Q；T128N和P129A）以提高FⅩ活化速率并延长效应时间，其Ⅱ期试验表明以每日1次的频率皮下给药对合并抑制物患者可起到较好的预防作用[16]。

二、非凝血因子类药物

非凝血因子药物治疗血友病的思路有两个，一种是直接模拟血浆中活化凝血因子功能，实现非因子替代效果；另一种是通过抑制体内抗凝物质如抗凝血酶（AT）、组织因子途径抑制物（TFPI）、活化蛋白C（APC）及蛋白S（PS）等使促凝和抗凝再平衡，增加凝血酶的生成[17]。

（一）非因子替代治疗药物

Emicizumab是一种经修饰的人源化双特异性抗体，通过模拟FⅧa辅因子功能，在磷脂膜表面同时桥接FⅨa及FⅩ，活化FⅩ从而恢复凝血平衡，其长达30 d的半衰期允许每1、2、4周用药1次，可将HA表型由重度转为轻度，用于伴或不伴抑制物HA患者的常规预防治疗[18]–[19]。Callaghan等[20]汇总分析了401例既往参与Emicizumab系列临床试验受试者的数据，发现HA患者ABR随着Emicizumab预防性使用而持续下降并维持在较低水平（需治疗的ABR 24周后<1），大多数患者靶关节出血得到有效控制，患者对Emicizumab依从性良好，在长期观察中未出现新的安全问题。这项研究肯定了长期应用Emicizumab预防HA出血的疗效及安全性，但局限性在于亚洲受试者样本数据较少。在此基础上，杨仁池等[19]开展的HAVEN5研究旨在评估Emicizumab在亚太HA患者中的有效性、安全性、免疫原性和药代动力学（PK）特征，将纳入的70例HA患者（重型67例，中间型3例）随机分为3组，分别予Emicizumab 1.5 mg/kg每周预防（29例）、6.0 mg/kg每4周预防（27例）及无预防（14例），与无预防组相比，两个预防组的受试者获得了稳定的平均药物血浆谷浓度和有效的出血控制（需治疗的ABR降低>95％），无血栓事件及血栓性微血管病（TMA）发生。这一研究展现了Emicizumab在亚太地区HA患者中良好的效能及安全性，为我国血友病预防治疗模式转变提供了有力的证据。近日，有关Emicizumab在轻中型HA患者中预防治疗的Ⅲ期研究HAVEN6结果已公布，此研究中位随访时间为55.6周，72例不伴抑制物的非重型HA受试者在接受Emicizumab用药后需治疗的ABR为0.9，整体安全性可控，无任何不良事件导致停药、治疗中断或死亡的报道，发生的1例血栓事件与用药无关[21]。此研究证实了Emicizumab预防轻型和中间型HA患者出血的有效性，其应用有望降低非重型患者产生抑制物的风险，但仍需更大量样本数据以监测其可能导致的血栓事件。Emicizumab在既往未接受治疗的<1岁婴幼儿中的预防作用和安全性研究HAVEN7正在进行，以期扩展在血友病患者中的适用人群。Emicizumab不适用于HB患者，其对骨关节健康的长期作用有待进一步观察。尽管目前观察到的抗Emicizumab抗体发生率较低，但部分中和抗体的出现会阻碍其作用或加速其清除，对于用药后疗效不佳的患者应尽早评估干预[22]–[23]。

在近年的研究中，新一代FⅧa功能模拟药物Mim8使HA患者血浆血凝块形成正常化，在体外样本及体内HA小鼠实验中其作用效力比同序列Emicizumab类似物高约15倍，这一变化主要与Mim8抗FⅨa臂加强FⅨa蛋白水解活性相关[24]。Mim8在HA患者中作用的早期试验FRONTIER1（NCT04204408）正在进行。

（二）止血再平衡疗法

1. TFPI抑制：TFPI是针对外源性凝血途径的抗凝物质，在凝血的起始阶段可结合并抑制FXa和TF/FVIIa复合物的活性，对于FⅧ或FⅨ不同程度缺乏的血友病患者而言，阻滞TFPI有助于增加FXa的生成以恢复出凝血平衡[25]。针对TFPI靶向抗体药物包括Concizumab、Marsticimab和MG1113等，其主要靶点为TFPI的K2区[26]–[28]。已有数据表明每日1次Concizumab皮下注射对于伴抑制物的HA及HB患者（Explorer4）和重型HA患者（Explorer5）是一种耐受性和药效良好的治疗方式，具有长期（至少76周）预防出血的作用[26],[29]。在2023年3月，Concizumab获得加拿大批准用于伴FⅨ抑制物并需要常规预防性治疗的青少年和成年HB患者[30]。在Marsticimab的Ⅰb/Ⅱ期研究[31]中，完成试验的受试者无论自身是否存在抑制物，以每周1次的频率皮下注射Marsticimab后自身ABR均有明显下降，全体受试者平均ABR为2.67且各亚型间无明显差别；注射部位反应为最常见的不良事件，血栓以及治疗相关的严重不良事件均未报道。Marsticimab的Ⅱ期研究提供了长达1年的用药数据，该药展现出的安全性、疗效以及患者耐受性同Ⅰb/Ⅱ期研究基本一致[32]。目前，Marsticimab的Ⅲ期BASIS试验（NCT03938792）已达到其主要终点，与按需使用凝血因子相比出血率降低92％，相较于预防性使用凝血因子ABR下降35％。Marsticimab一个突出特征是用药剂量固定而不受体重影响，其优势为适用于HA及HB患者且不受抑制物影响，该药物在我国的Ⅰ期研究已完成[33]。受既往试验中HB患者数较少的影响，Marsticimab对HB的治疗作用仍需进一步评估，其整体安全状况及在血友病患儿中的应用有待继续研究。MG1113已在血友病动物模型中起到了恢复凝血酶生成与减少出血的作用，该药在血友病患者中的应用信息仍然缺乏[28],[34]。

2. AT沉默：AT属丝氨酸蛋白酶抑制物家族成员，主要由肝细胞合成，是重要的抗凝物质，与凝血途径中的丝氨酸蛋白酶特别是凝血酶、FⅩa、FⅨa共价结合发挥抗凝作用。Fitusiran是一种小干扰RNA（siRNA），其作用机制为特异性结合并降解肝细胞内AT信使RNA（mRNA），使AT基因表达沉默从而降低循环中AT水平，增加凝血酶的产生，这一作用呈剂量依赖性且已在一项无抑制物的Ⅰ期试验中得到了验证[35]。Pasi等[36]报道了Fitusiran一阶段研究的另一项成果：17例有抑制物的HA或HB患者以每月1次的频率接受3次50 mg（6例）或80 mg（11例）固定剂量Fitusiran皮下注射，在112 d的过程中，Fitusiran耐受性和整体安全性良好，用药后受试者出血次数均减少且其中11例（64.7％）未发生出血事件，最常见的不良反应为注射部位红斑，出现的肝酶升高整体表现为轻度且持续时间短暂。在此基础上，Fitusiran的二阶段开放标签延长研究（OLE）以及第三阶段ATLAS系列试验同样取得进展，在疗效方面，OLE显示预防性应用Fitusiran可将受试者的ABR（中位值0.84）长期控制在一个较低水平；两项随机化ATLAS试验显示不管是否存在抑制物，以每月1次间隔予Fitusiran 80 mg预防治疗后受试者ABR均较按需治疗组降低约90％，实现无出血的中位水平，这对于改善健康相关生活质量是有意义的[37]–[39]。该药有望使HB患者获得预防性保护，其引发血栓事件的可能性仍应引起重视。

3. PS抑制：PS由PROS1基因编码，在抗凝中发挥多重作用，作为APC的辅因子参与FⅤa与FⅧa的灭活，也是TFPI的辅因子，其自身尚可直接阻碍凝血酶原酶复合物，因而靶向抑制PS可能促进凝血再平衡[40]–[41]。研究者们发现经PROS1基因沉默或PS蛋白抑制处理的血友病小鼠凝血功能得到改善，关节内出血和膝关节肿胀均显著减轻，对血友病关节病起到保护作用[41]。靶向PS有望成为血友病治疗的新方法，其可行性及安全有效性亟需临床研究结果进一步分析。

三、血友病药物治疗存在的问题

不同于基因治疗，血友病的药物治疗无法使机体持久产生凝血因子或永久达到出凝血平衡，受机体代谢影响无法做到一次给药治愈该病。对于凝血因子药物而言，尽管不少制剂半衰期及给药间隔均较前延长，但大多数仍需经静脉途径反复输注，存在产生抑制物的风险，影响患者依从性，如所获凝血因子谷浓度较低，则关节损害无法避免[1]。对于非因子类药物而言，最突出的问题为患者自身存在易栓及高凝因素或因突破性出血需联用止血药从而引发出凝血失衡形成血栓。Concizumab研究曾在2020年3月一度中止，原因是Ⅲ期试验中（Explorer7和Explorer8）3例患者发生5次非致死性血栓事件（动脉2例次，静脉3例次），经分析发现这些患者平日即有血栓风险并在事件发生当天使用了止血药物。在Fitusiran的Ⅱ期研究中曾出现患者因创伤后出血采用标准剂量FⅧ替代治疗后静脉窦血栓致死的案例[17],[42]。在遭遇突破性出血时应以多大剂量联用其他止血剂以尽可能平衡出血和血栓之间的矛盾是该类药物应用必须面对的挑战[17],[43]。另一项研究显示，与EHL FⅧ有关产品相比，Emicizumab的出血性事件报告率较低，而血栓性不良事件报告比率较高[44]。对于Emicizumab而言应尽量避免使用凝血酶原复合物（PCC）以防止TMA，对于肥胖、吸烟、既往深静脉血栓形成等伴血栓形成风险的患者在联合应用rFⅦa过程中应格外警惕非ST段抬高性心肌梗死及肺栓塞的发生[45]–[46]。此外，使用非因子药物的患者，其体内生理性的凝血因子仍处于缺乏的状态，长期应用可能会对骨关节健康有影响，需定期随访及密切监测以防止关节病变发生[1]。

综上，血友病替代治疗的飞速进步有助于尽早使患者获得个体化预防治疗，真正提高其生存质量，为难以进行基因治疗的患者提供了良好解决方案。从被动输注血浆制品或标准半衰期凝血因子到主动应用半衰期较前延长的凝血因子制品及非因子类药物，该病多年来在治疗过程中遇到的难题被逐一破解，这一疾病的高水平防治已不再遥不可及。
